# Cohen syndrome combined with psychiatric symptoms: a case report

**DOI:** 10.1186/s12888-024-05626-1

**Published:** 2024-03-04

**Authors:** Xinming Li, Sufang Qi, Wenjie Li, Xin Liu, Zhicheng Xue, Tiangui Yu, Guanglei Xun

**Affiliations:** Shandong Mental Health Center, Shandong University, Mail Code: 250014, 49# Wenhua Eastern Road, Jinan, Shandong Province P.R. China

**Keywords:** Cohen syndrome, Psychiatric symptoms, Gene mutation

## Abstract

**Background:**

Cohen syndrome (CS) is a rare autosomal recessive inherited condition characterized by pathological changes affecting multiple systems. The extensive clinical variability associated with CS poses a significant diagnostic challenge. Additionally, there is limited documentation on the co-occurrence of CS with psychiatric symptoms.

**Case report:**

We report a case of a 30-year-old patient exhibiting characteristic physical features and psychiatric symptoms. Whole exome sequencing identified two heterozygous variants, a nonsense variation c.4336 C > T and a missense mutation c.4729G > A. Integrating clinical manifestations with genetic test results, we established the diagnosis of CS combined with psychiatric symptoms.

**Conclusions:**

This case introduces a novel missense variant as a candidate in the expanding array of *VPS13B* pathogenic variants. Its clinical significance remains unknown, and further investigation may broaden the spectrum of pathogenic variants associated with the *VPS13B* gene. Early diagnosis of CS is crucial for the prognosis of young children and holds significant importance for their families.

## Introduction

Cohen syndrome (CS), an autosomal recessive condition caused by a single gene, was initially characterized by Cohen and colleagues in 1973 [1]. This hereditary disorder is exceptionally rare globally [2]. Patients with CS from Finland exhibit more homogeneity compared to those from other countries, where a greater degree of clinical variability is observed [3]. Extensive clinical variability has made the diagnosis of CS a real challenge. Studies have indicated that CS encompasses pathological changes across multiple systems during periods of growth and development [4], including craniofacial abnormalities (microcephaly, low hairline and short philtrum), dentition issues (prominent upper incisors, extensive alveolar bone loss), ophthalmological manifestations (constricted visual fields and nyctalopia), hematological abnormalities (leukopenia), musculoskeletal system involvement (ligamentous laxity and articular hypermobility), neurological features (brisk tendon reflexes and muscular hypotonia), among others [3, 5–7].

CS is linked to gene mutations, with the vacuolar protein sorting 13 homolog B (*VPS13B*, also referred to as *COH1*) gene currently recognized as the exclusive gene associated with CS [8]. *VPS13B* plays a crucial role in preserving Golgi integrity, Golgi protein glycosylation, and endosome-lysosome transport [9, 10]. Genetic mutations impact the structure and function of proteins, resulting in manifestations of the disease across multiple systems. Variations in mutation locations contribute to the observed clinical heterogeneity among affected individuals. CS encompasses over 200 pathogenic variants, with missense and nonsense variants being the most prevalent [4].

The majority of previously reported cases involve pediatric patients with CS. In this study, we present a unique case involving a 30-year-old patient with CS combined with psychotic symptoms. Additionally, a novel candidate missense variant was identified through genetic testing.

## Case presentation

A 30-year-old female, currently under psychiatric medication, sought fertility counseling at our outpatient service.

The patient experienced a difficult birth, and her early years were marked by frailty, leading to frequent colds during childhood. Various aspects of her growth and development significantly lagged behind her peers. During childhood, she exhibited hypotonia and motor developmental delays, including challenges in rolling over at 6 months old, crawling at 11 months old, standing at 18 months old, and walking at 24 months old. Her parents noted a distinct softness in their daughter’s body when held. Furthermore, the patient exhibited intellectual challenges, despite concerted efforts by both parents to aid her studies, resulting in consistently low academic performance. As the patient continued to age, she faced ongoing difficulties in contributing to household chores. Additionally, her vision deteriorated, particularly at night. Throughout the course of growth and development, the patient progressively exhibited central obesity.

In 2010, the patient experienced difficulties sleeping and exhibited psychiatric symptoms. These symptoms included reciting poems independently, being difficult to interrupt, engaging in laughter or conversations with unseen entities, and showing a lack of response to family inquiries. Additionally, she frequently wandered aimlessly, colliding with obstacles without an apparent awareness of how to navigate around them. Consequently, her parents sought assistance at a psychiatric outpatient clinic, where she received a diagnosis of schizophrenia. Following months of olanzapine treatment, the patient’s psychiatric symptoms resolved. In 2014, the patient was diagnosed with diabetes, with her highest blood sugar level recorded at 21mmol/L. She married in the same year. However, in the latter part of 2015, she discontinued medication due to pregnancy and gradually experienced a recurrence of certain psychiatric symptoms. Similar to her initial episode, the patient experienced severe sleep disturbances. Additionally, she engaged in incomprehensible conversations with unseen entities. At times, she would cry intensely due to an inexplicable sense of being targeted, only to laugh without apparent cause shortly afterward. Her temper became increasingly irritable, leading to instances of throwing objects and occasional aggression towards family members. Tragically, she experienced a stillbirth during her first pregnancy at 5 months. In the first half of 2016, the patient was admitted to our hospital. A routine brain CT scan revealed no organic changes. Following a comprehensive treatment regimen including olanzapine, magnesium valproate, and other medications, her psychotic symptoms gradually ameliorated, and her mood stabilized. In 2018, she gave birth to a baby girl, albeit one month premature. The child, now four years old, demonstrates development in intelligence, motor skills, and language on par with her peers.

In consideration of plans for another child, the patient sought fertility counseling from our facility. During the outpatient clinic examination, our attention was drawn to the distinctive physical characteristics of the patient, who walked with a stiff gait. She presented with abnormal features, including central obesity with slender limbs and a prominent buffalo back. Facial attributes encompassed microcephaly (head circumference of 50 cm), a low hairline, flat occiput, irregular dentition, high-arched palate, short philtrum, and limited mouth opening. Additionally, the patient exhibited the simian line, substantial finger gaps, poor finger fine movement, and an inability to straighten her slender fingers well. Furthermore, the patient’s speech was unclear with limited language expression, and she displayed mental retardation, capable only of performing basic additions and subtractions. Her intelligence quotient (IQ) score on the Wechsler Adult Intelligence Scale-Revised in China (WAIS-RC) was at the borderline level, measuring 75. The patient has a younger brother who exhibits a similar condition. Her parents and other relatives are free from the disease.

Upon reviewing the patient’s historical and recent laboratory results, we extracted information revealing that the neutrophil count ranged from 2.29*10^9 to 4.56*10^9 (reference range: 4*10^9–10*10^9), the neutrophil ratio ranged from 29.9 to 58.8% (reference range: 50-70%), and glucose levels fluctuated between 7.3 and 12.0 mmol/L (reference range: 3.6–6.1). The remaining components of the complete blood count appeared to be within normal ranges.

**Fig. 1:**
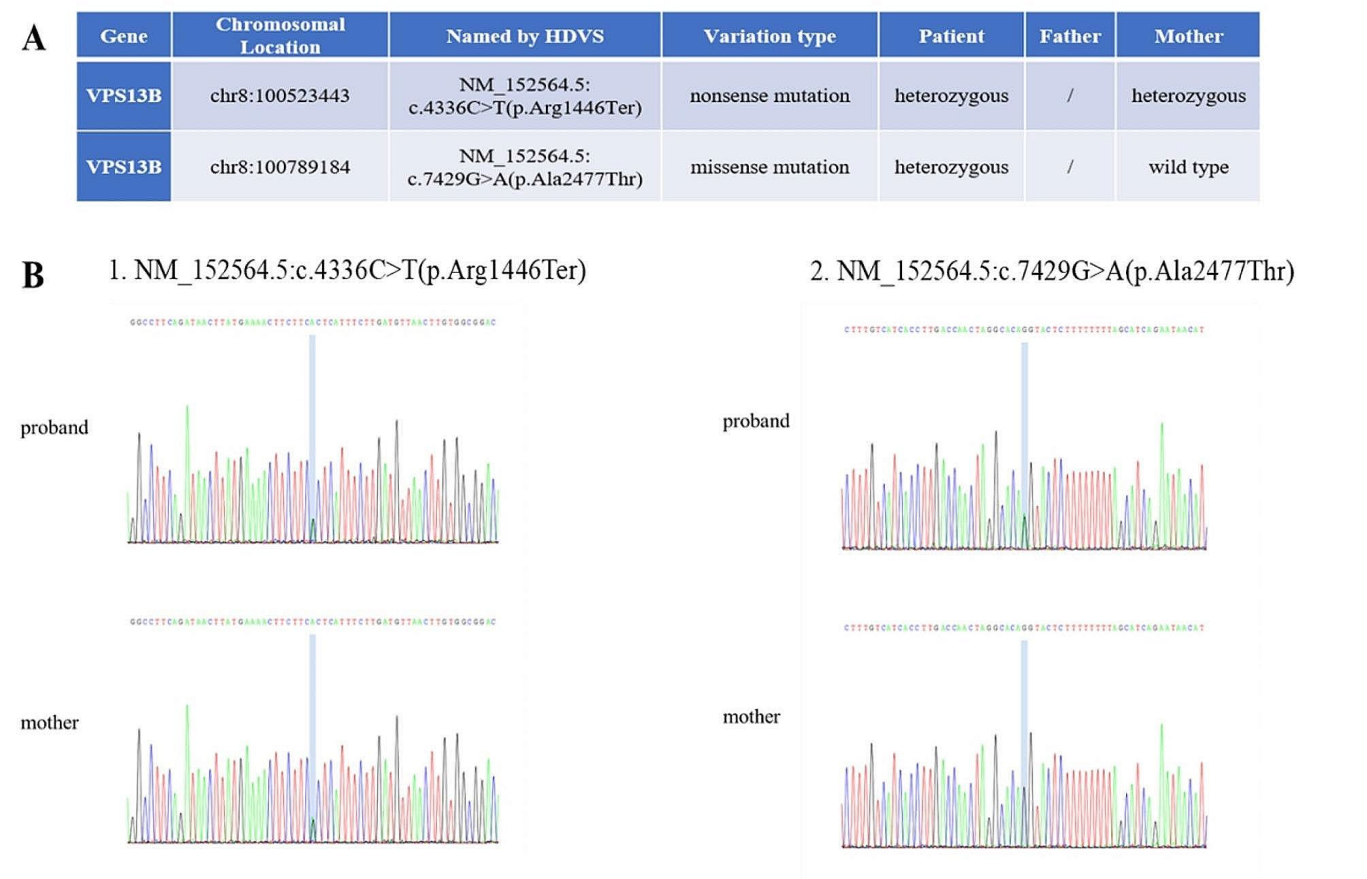
The patient’s genetic test results. **A** Variation associated with the clinical phenotype of the proband. **B** The patient’s Sanger sequencing test results

Based on the patient’s condition, we recommended genetic testing for developmental disorders. Following the informed consent obtained from both the patient and her mother, we collected 2 milliliters of venous blood from each participant. The blood samples were then sent to a commercial company for genetic testing related to intelligence and motor developmental delay diseases. The proband’s genes were identified through whole-exome sequencing and the subsequent results revealed two heterozygous mutations in the exon region of *VPS13B*: c.4336 C > T, identified as a nonsense mutation, and c.4729G > A, identified as a missense mutation. Sanger sequencing confirmed the presence of the heterozygous variant *VPS13B* c.4336 C > T in the proband’s mother (Fig. 1A, B). We attempted to reach out to the patient’s father and brother for genetic testing; however, they declined to participate, limiting the availability of additional information.

## Discussion

The patient in this article presents indications of several disorders that warrant acknowledgment. The central obesity observed in the patient is reminiscent of Cushing syndrome. However, the absence of hypokalemia, a full moon face, edema, or hypertension, coupled with the patient’s distinctive developmental characteristics and inconclusive laboratory test results, does not support a diagnosis of Cushing syndrome. The patient exhibits certain features such as psychotic symptoms, language and intellectual disabilities, but lacks a narrow range of interests, stereotyped motor behaviors, and social interaction disorders, leading to the exclusion of an autism spectrum disorder diagnosis. Kolehmainen et al. proposed eight clinical measures (developmental delay, microcephaly, typical Cohen syndrome facial gestalt, truncal obesity with slender extremities, overly sociable behavior, joint hypermobility, high myopia and/or retinal dystrophy, and neutropenia) to evaluate CS, and patients meeting six or more criteria could receive a diagnosis [11]. According to these criteria and the relevant gene testing results, the patient in question has met the diagnostic criteria for CS. Furthermore, the patient had previously been misdiagnosed with schizophrenia. Considering her history of delayed intelligence and developmental disability, in accordance with the International Classification of Disease 11th Revision (ICD-11), the diagnosis of CS (LD90.Y) combined with psychiatric symptoms is deemed appropriate.

In general, diagnosing CS in young children presents a significant challenge, as most cases are identified after the age of 6 [12], with the patient discussed in this article receiving her diagnosis at the age of 30. The delayed diagnosis is attributed to the ambiguous onset of certain clinical signs [13, 14]. Research by El et al. on CS facial features indicates common characteristics, such as a short neck and a square face with micrognathia and full cheeks, between the ages of 2–6, potentially offering early diagnostic clues [15]. Facial features in CS patients become more distinct with age, and the enlargement of the corpus callosum on early brain MRI can be indicative of CS [3]. Trunk obesity is a prevalent feature in CS cases, leading to the term “abnormal truncal fat distribution” as an alternative to “obesity” due to enlarged waist circumference but a normal body mass index [16]. Previous studies suggest that preadipocytes lacking *VPS13B* may exhibit an increased tendency to differentiate into fat-storing cells, contributing to excess fat accumulation in CS patients [16]. Dysregulated insulin response due to defective *VPS13B* may explain the occurrence of diabetes in some CS patients [16]. However, this case does not rule out the potential relationship between diabetes and olanzapine use. Additionally, as observed in our case, intermittent granulocytopenia can occur in CS patients, though it is generally non-fatal [17]. Retinopathy in CS exhibits an age-related relationship, with some patients experiencing gradual visual deterioration over their lifetime [4]. In this case, the patient had normal vision during childhood and gradually developed amblyopia and night blindness with age. However, the patient did not undergo a professional eye examination. Unfortunately, effective methods to halt the progression of certain visual impairments in CS patients are currently unavailable [18].

In this case, the genetic analysis performed on this patient included the whole exome, mitochondrial genome, copy number variation, and exon regions of clinical significance. Whole exome sequencing conducted high throughput sequencing of 180,000 exons of about 25,000 genes in the whole genome of the proband blood DNA samples, and found that the variants that may be related to the clinical phenotype of the proband were located on the *VPS13B*, *GRIA4*, and *ATRX* genes, respectively. In this study, whole-exome sequencing identified heterozygous mutations in the *VPS13B* gene of the proband, including one reported nonsense mutation NM_152564.5: c.4336 C > T (p.Arg1446Ter) and one novel missense mutation NM_152564.5: c.7429G > A (p.Ala2477Thr). The reported mutation is considered potentially pathogenic according to ACMG principles (LP-PvS1 + PM2Supporting), while the novel missense variant is categorized as having unknown clinical significance (VUS = PM2_Supporting + PP3), as it has not been reported in literature or public databases. The protein coded by the *VPS13B* gene plays a crucial role in synaptic growth. Mutations in the gene can lead to inhibition of synaptic growth, giving rise to various clinical characteristics. Among these, the most prominent is the nervous system phenotype, encompassing progressive retinal dystrophy, non-progressive intellectual disability, and microcephaly [19]. Prior genetic studies linked 16p13.11 duplication and *VPS13B* deletion to schizophrenia and mental disorders [20]. The pathogenicity of the missense variation found in this patient warrants further clarification through functional experiments.

## Conclusions

In this study, we reported a case of a CS patient with psychotic symptoms. Whole exome sequencing revealed a novel missense variant in the *VPS13B* gene within a CS family, thereby expanding the growing catalogue of disease-causing variants in *VPS13B*. Additionally, in this case, we meticulously adjusted the patient’s medication regimen, advised her to undergo a comprehensive ophthalmic examination, and instructed her to undergo regular blood glucose, lipid tests, and pregnancy examinations based on her current condition. Furthermore, we recommended genetic testing for developmental disorders for her husband to rule out the presence of mutated genes.

In summary, from a clinical perspective, genetic testing can assist individuals with distinctive appearances and developmental disorders in excluding inherited metabolic issues. Early diagnosis of CS is crucial for the prognosis of young children and holds significant importance for their families. Given that CS patients often experience multi-systemic involvement, early multidisciplinary intervention can reduce the incidence of complications. Early intervention programs, encompassing specialized education and enhanced supervision to bolster daily living skills, play a crucial role in enhancing patients’ quality of life.

## Data Availability

The data used during the current study are available from the corresponding author on reasonable request.

## Abbreviations

CS: Cohen Syndrome
*VPS13B*: Vacuolar Protein Sorting 13 Homolog B
IQ: Intelligence Quotient
WAIS-RC: Wechsler Adult Intelligence Scale-Revised in China
ICD-11: International Classification of Disease 11th Revision
MRI: Magnetic Resonance Imaging
